# Methodological and clinical implications of a three-in-one Russian doll design for tracking health trajectories and improving health and function through innovative exercise treatments in adults with disability

**DOI:** 10.1186/s12874-018-0480-3

**Published:** 2018-03-14

**Authors:** James H. Rimmer, Cassandra Herman, Brooks Wingo, Kevin Fontaine, Tapan Mehta

**Affiliations:** 1 Department of Occupational Therapy, School of Health Professions, University of Alabama at Birmingham, 1716 9th Ave. South, Birmingham, AL 35294-1212 USA; 2 Department of Health Behavior, School of Public Health, University of Alabama at Birmingham, 1665 University Boulevard, Birmingham, AL 35294-0022 USA; 3 Department of Health Services Administration, School of Health Professions, University of Alabama at Birmingham, 1716 9th Ave. South, Birmingham, AL 35294-1212 USA; 4 UAB-Lakeshore Research Collaborative, School of Health Professions, University of Alabama at Birmingham, 1716 9th Ave. South, Birmingham, AL 35294-1212 USA

**Keywords:** Neurologic disability, Exercise, Exercise rehabilitation, Health, Longitudinal

## Abstract

**Background:**

Hybrid research designs targeting adults with neurologic disability are critical for improving the efficiency of models that can identify, track and intervene on identified health issues.

**Methods:**

Our Russian doll framework encompasses three study phases. Phase 1 involves prospectively following a cohort of participants with disability to examine the relationships between rates of health and functional deficits (e.g., pain, fatigue, deconditioning), functional measures (e.g., cardiorespiratory endurance, strength, balance), and environmental and sociocultural factors. In Phase 2, eligible participants with neurologic disability from Phase 1 (in our example, individuals with multiple sclerosis) are screened and randomized to a clinical exercise efficacy trial. In Phase 3, study participants are enrolled in a home-based teleexercise trial to test the feasibility and replicability of delivering the clinical exercise study in the home.

**Discussion:**

This unique three-in-one Russian doll framework serves as a foundation for informing and guiding researchers and clinicians in treating certain health and functional deficits in people with neurologic disability using exercise as a primary treatment modality in both the clinical and home settings. It offers a unique perspective for understanding the critical issues of functioning, health maintenance and quality of life for people with neurologic disability across a longitudinal framework.

**Trial registration:**

Study 2 ClinicalTrials.gov identifier NCT02533882 (retroactively registered 03/06/2015). Study 3 ClinicalTrials.gov identifier NCT03108950 (retroactively registered 04/05/2017).

## Background

Health trajectories in the general population typically impacted by lifestyle behaviors and genetics have a third, less understood dimension in people with disabilities: the onset and course of secondary conditions and their ‘weighted’ or ‘additive’ effect on health and function [1]. Several studies have reported that the highest rates of secondary conditions among people with neurologic disability include pain, fatigue, deconditioning (weakness), cardiometabolic disease, obesity and emotional distress including depression and anxiety [2–13]. These conditions impose substantial limitations in rates of participation in general life activities including employment, social engagement and performing instrumental activities of daily living [9, 13–18]. Identifying and/or developing effective methods and strategies for preventing and treating these debilitating health conditions continues to be an important priority in disability and health research [19–21].

One extremely important area of treatment need in people with neurologic disability is exercise [22]. In the general literature on people without disabilities, regular exercise has been shown to reduce the risk of chronic diseases and improve overall health, function and quality of life [20, 23, 24]. Unfortunately, preventive exercise to treat and manage secondary health conditions associated with neurologic disability continues to be underutilized for several reasons. First, many of the conditions associated with neurologic disability including joint pain, fatigue, weakness, balance, sensory impairments, anxiety, depression and spasticity [25, 26] limit opportunities to engage in common forms of exercise and lead to higher levels of sedentary behavior [8, 27–29]. Second, high rates of unemployment or underemployment among people with neurologic disability [15, 30–32] limit general overall activity across the day increasing their risk of sedentary behavior. Third, limited ability to walk outdoors (the most common form of physical activity in the general population) due to difficult terrain, safety, or the inability to walk [33] present less options for obtaining regular exercise. And fourth, limited public and private transportation to and from community fitness facilities presents a significant challenge in attending these community-based exercise programs [34–36]. In cases where individuals with neurologic disability are able to get to an exercise facility, they often find that much of the equipment is inaccessible and the program staff is unable to accommodate their specific health and functional needs [37–39].

The lack of prospective, epidemiological data that addresses a broad spectrum of secondary health conditions and their impact on individual health and function in people with neurologic disability limits the ability of researchers, clinicians, practitioners and policymakers to prioritize treatment strategies and interventions for this population. It is also makes it difficult to determine how certain health behaviors or factors (e.g., diet, exercise, medications, use of assistive technology, employment) impact secondary conditions individually or in combinations, and how environmental factors may play a role in the onset and severity of secondary conditions [40]. Detecting the risk of progression at an earlier stage and implementing effective interventions to reduce their potential adverse effects on health and function is a critically needed area of research.

To address these deficits, we developed a three-in-one Russian doll framework with three phases: a) exploratory phase: advance understanding of the onset and course of secondary health conditions in people neurologic disability in relationship to exercise and environmental factors; b) efficacy phase: determine the impact of specific types and doses of exercise in improving health and function in certain underperforming systems (e.g., cardiorespiratory, musculoskeletal, neuromotor) in adults with neurologic disability; and c) effectiveness/scale-up phase: rapidly translate successful clinical exercise trials into the home setting using telehealth technology that has growing potential to reach geographically (e.g., rural) and socioeconomically (e.g., transportation barriers) isolated disability groups.

## Methods/design

### Three-in-one ‘Russian doll’ study design

**Fig. 1:**
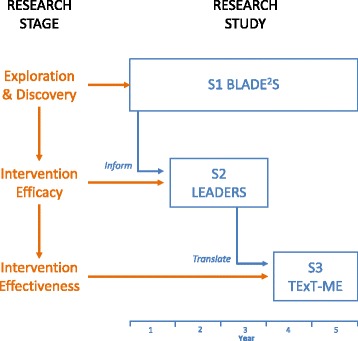
Three-In-One ‘Russian doll’ Study Design. BLADE^2^S (S1) – Birmingham-Lakeshore Aging with Disability Exercise and Environment Study. LEADERS (S2) – Lakeshore Examination of Activity, Disability and Exercise Response Study. TExT-ME (S3) – Telehealth Exercise Training for Monitoring and Evaluation

Our Russian doll study implementation framework is based on a *nested trial* design with the subsequent study phases of efficacy and effectiveness nested within the overarching exploratory phase (Fig. 1). The first study (S1) is a prospective, longitudinal cohort study that is examining the health and function trajectories of adults with neurologic disability (multiple sclerosis, stroke, spinal cord injury, mild traumatic brain injury, Parkinson’s). Data allow us to examine differences in underperforming physiologic and neuromotor systems (e.g., low cardiorespiratory reserve, musculoskeletal weakness, balance impairments) and to develop interventions that address these deficits.

Participants who enroll in the exploratory study (S1) are randomly assigned to either an efficacy exercise trial (S2) or waitlist control group (S3). After the clinical exercise trial is completed, waitlist controls participate in a home-based translational exercise effectiveness trial (S3) that replicates the successful elements identified in S2 using telehealth technology to monitor the intensity and safety of exercise. All participants continue their enrollment in the prospective cohort study (S1) before and after the interventions. The design is also ethical because every participant receives a desirable treatment (i.e., exercise) in the community or home setting.

### Setting

The exploratory (S1) and clinical efficacy studies (S2) are conducted in a state-of-the-art, universally designed community health and fitness facility. The facility was specifically designed to address standard access issues typically found in fitness centers. All areas of the facility (e.g., lockerrooms, bathrooms, parking, fitness center equipment, etc.) are completely barrier-free. The translational study (S3) is conducted in the home setting.

### S1: Birmingham Lakeshore Aging with Disability Exercise-Environment Study (BLADE^2^S)

#### Design

BLADE^2^S is a multidisciplinary, prospective epidemiologic longitudinal study examining the influence and interaction of neurologic disability on general health and secondary health conditions (e.g., pain, fatigue, weakness, depression, cardiovascular decline), functioning, and quality of life in adults with neurologic disability 18 years and older. BLADE^2^S has four aims: (1) evaluate the health trajectories within and between groups using norm-referenced national data sets; (2) identify associations between a range of variables (e.g., socioeconomic, lifestyle, behavioral) and health, function and quality of life outcomes; (3) investigate the effects of changes on study variables on health, function and quality of life outcomes; and (4) provide a data-driven framework for the development and testing of interventions that address secondary health conditions, disability-specific physiological/neuromotor functional deficits, and quality of life.

#### Recruitment

Participants are recruited into BLADE^2^S from the surrounding community using a variety of recruitment techniques including flyers, mailings, attending community events, word-of-mouth, and physician referrals. Participants are eligible for inclusion if they are 18 years of age or older at the time of the phone screen and considered safe to exercise by their personal physician. Participants complete a phone screen prior to enrollment identifying a primary disability or condition, age, race, and gender. Participants must also be willing to come for in-person visits one time per year and complete questionnaires via mail every 6 months. Eligibility for the clinical efficacy (S2) and home-based translational studies (S3) is also assessed through the phone screen. Studies designed to assess the feasibility or efficacy of exercise interventions often require additional inclusion and exclusion criteria. Additional screening measures (e.g., stable heart condition, blood pressure and body weight) ensure no contraindications to exercise.

#### Measurements

Components of the annual BLADE^2^S in-person assessment and evaluation are shown in Table 1 (a detailed description of each test protocol is in a manual of procedures and is available upon request). The following variables are being collected via questionnaire: age, race, and sex of the participant, medical history, state of menopause (if female), preventative screening for both male and female (breast exam, pap test, mammogram, prostate exam), cigarette smoking (including smoking status, pack-years exposure), alcohol intake, physical activity level, general health, access to care, insurance status, marital status, measures of socioeconomic status (education and income), social support, and psychosocial factors (e.g., depressive symptoms, anxiety, loneliness, fatigue). Upon completion of the study questionnaires, a staff member and participant review the responses to ensure accuracy.

**Table 1:** Assessment Measures

Domain	Selected Measures & Tests
*Health*	
Anthropometrics	Height, Weight, Waist Circumference, BMI, Body Composition (DXA)
Health Biomarkers	Blood Pressure, Fasting Glucose, Lipids, Insulin
*Physical Function*	
Strength	Grip Strength, Biodex™ Closed Chain Push/Pull (upper body), Biodex™ Knee Flexion/Extension
Balance	Biodex™ Balance Limits of Stability, Timed Up and Go*, Repeated Chair Stands^a^
Cardiorespiratory Function	Submaximal VO_2_
Gait	Walking Velocity (GaitRite® Mat)^a^
*Self-Report Measures*	
	PROMIS Questionnaires^b^
	3-Item Loneliness Scale
	Nutrition Self-Efficacy
	Physical Exercise Self-Efficacy
	Godin Leisure Time Exercise Questionnaire
	Lakeshore Facility Utilization^c^

^a^Tests that require standing/walking are only performed with ambulatory participants

^b^Patient Reported Outcomes Measurement Information System – pain intensity, pain interference, fatigue, anxiety, depression, physical function with mobility aid, ability to participate in social role and activities, sleep disturbance

^c^Questionnaire developed specifically for Lakeshore Foundation

Participants also undergo a series of tests and procedures to assess their health and physical function. Health is assessed by measurement of anthropometrics (height, weight, waist circumference, body composition [via Dual-energy X-ray absorptiometry]; DXA) and health biomarkers (e.g., blood pressure, fasting blood glucose, insulin and lipids). Physical function is assessed through measurements of strength, fitness, balance, and cardiorespiratory endurance. All tests and procedures are conducted by research staff under the supervision of the study coordinator. Participants complete all testing across a two-day testing period. Anthropometric measurements, gait, balance, and strength testing are conducted on day 1, and fasting blood draw, submaximal cardiorespiratory endurance and additional balance measures are collected on day 2. Participants complete a questionnaire packet to assess a variety of health domains such as sleep, fatigue, pain, anxiety, and depression between assessments 1 and 2. The entire assessment takes approximately 3 h to complete.

#### Personnel training and quality control

All research staff are carefully trained and closely monitored for sensitivity to the attitudes, abilities, and limitations of the participants. Staff performance is continuously monitored by supervisors, and team meetings are held by the research coordinator weekly to discuss frequently asked questions and to resolve unusual circumstances. Feedback to staff is provided at periodic intervals.

#### Data management

Each participant is identified by a unique confidential study number. Data from the self-report and physiological/neuromotor assessments are either scanned or double entered by data management staff. Data are stored on a dedicated database server, which is password protected, behind a firewall and backed up daily. All research data are stored in relational databases, which, among other features, allows for maintaining data integrity for accuracy and consistency across large data sets into which new results are constantly being added as they are collected.

### S2: Lakeshore Examination of Activity, Disability and Exercise Response Study (LEADERS)

#### Design

S2 studies in our Russian doll framework are referred to as LEADERS trials. These studies are conducted in a controlled setting and are efficacy trials designed to examine and compare responses to different types of exercise interventions for participants enrolled in BLADE^2^S (S1). After completing assessments, participants become part of a large pool of participants who are eligible for exercise interventions designed to address specific health and functional deficits. The studies are typically arranged by disability type. Researchers can propose various types of interventions for key target groups with neurologic disability.

#### LEADERS (S2) exercise efficacy trial for people with multiple sclerosis (MS)

An example of a study being conducted under our Russian doll framework is a comparative efficacy trial targeting people with MS. The research team determined that there was a lack of research on effective complementary and alternative exercise trials for people with MS and that, after testing in a controlled setting, could be replicated in the home environment [41, 42]. Many people with MS are not engaging in regular exercise (which includes short-term rehabilitation), and the risks associated with sedentary behavior include impaired neuromotor (e.g., reduced balance), musculoskeletal (e.g., weakness), cardiorespiratory (e.g., low energy reserve, fatigue) and mental (e.g., depression, anxiety) function [43].

The MS LEADERS study is comparing two innovative exercise treatments. Participants are randomized into one of three groups: (a) Intervention A – *Movement-2-Music* (*M2M*): combination of aerobic and strength training using music to enhance rhythm, cadence, enjoyment and adherence; (b) Intervention B - adapted yoga; and (c) waitlist control. A pre-post design is used to assess the contribution of exercise type, intensity and duration on a set of health and function outcomes. Primary health outcomes include self-reported pain, fatigue, and aspects of quality of life including loneliness, anxiety, and the ability to participate in social roles (NIH PROMIS questionnaires, see Table 1). Physical function outcomes include cardiorespiratory fitness, strength, balance, mobility and self-report physical function. All of these measures are a subset of the larger S1 BLADE^2^S longitudinal study and are repeated before and after the exercise trial. The interventions are 12 weeks in length (three sessions per week for 60 min.) and take place at a large exercise facility designed for people with disabilities.

The *M2M* group exercise classes are taught by trained dance instructors and are composed of a set of exercises tailored to the specific needs and capabilities of adults with MS. The class consists of several training components: a) warmup (10 min.) to increase range of motion; strength/balance (15–20 min.); aerobic conditioning (25–30 min. with rest periods as needed); cool down (5 min.).

The *adapted yoga* exercise classes are conducted by trained instructors based in the Iyengar approach to Hatha yoga and incorporates elements of other yoga practice. This approach allows for use of props (i.e., chairs, blankets, straps) to aid participants with limited flexibility and/or strength in obtaining poses, allowing for individual adaptations to be made for the target population [44].

During the intervention phase, the waitlist *control group* receives a biweekly newsletter containing health-related information to improve study retention. At the end of the study, controls receive the home-based exercise training intervention described below (S3).

#### Specific aims and hypotheses

MS LEADERS allows for investigation of the following aims and hypotheses:

Specific Aim 1: Examine the effects of two types of group exercise classes on the health and functional status of adults with MS.

##### Hypothesis 1.1:

Participants with MS participating in two different kinds of exercise interventions (M2M & adapted yoga) will evidence significantly greater gains in health status as measured by reductions in pain, fatigue, social isolation and increased quality of life (QOL) compared to waitlist care controls.

##### Hypothesis 1.2:

Participants in both exercise groups (M2M & adapted yoga) will obtain significantly greater gains in physical function as measured by improvements in cardiorespiratory fitness, strength, balance and flexibility compared to waitlist controls.

### S3: Telehealth Exercise Training - Monitoring and Evaluation (TExT-ME)

The growing awareness of successful clinical trials failing to reach end users has increased the visibility and importance of *knowledge translation*. Studies that remain in the clinic and never transfer into the home or community have limited applicability to people with neurologic disability who are in need of these programs. While the S2 LEADERS trials are critical for conducting high-fidelity interventions that quantify the effects of certain doses of exercise under the safest conditions, determining if these interventions can be conducted in real world settings is also extremely important for translating the science to the local level.

#### Design

The third and final study in our Russian doll framework is a translational home-based exercise trial established in S2 LEADERS using the latest innovative technology for delivering the intervention remotely. TExT-ME is designed to assess efficacious interventions in remote settings. Trials can be designed as feasibility or non-inferiority trials and can be delivered in a variety of contexts using a variety of different hardware and software products to fit the needs of specified interventions.

#### TExT-ME effectiveness trial for people with multiple sclerosis (MS)

MS participants in S2 LEADERS who were randomized to the waitlist control group are provided with the M2M or adapted yoga intervention based on findings from S2 LEADERS. The TExT-ME training and monitoring system is a user-centered design (UCD) involving a teleexercise coach (e.g., a trained research staff person) interacting remotely with a participant in their home via video conferencing applications. Participants wear monitors to maintain safe levels of exercise and provide feedback regarding exercise intensity to the instructors. Self-reported indicators of exercise intensity are also collected using the rating of perceived exertion (RPE) scale [45]. The training system includes three components: 1) Teleexercise monitoring station; 2) Exercise participant tablet application; and 3) Biometric/physiologic monitoring hardware (heart rate).

The teleexercise coach ensures that the exercise is safe and effective for the participant and also provides individual tailoring of the intervention if needed. The monitoring station includes multiple screens to simultaneously display 2-way video conferencing with participants and record data on exercise intensity and exercise response.

#### Specific aims and hypotheses

MS TExT-ME allows for investigation of the following aim and hypotheses:

Specific Aim: Examine the replicability and effectiveness of a home-based M2M teleexercise trial using the successful elements from the onsite clinical efficacy trial (MS LEADERS) on waitlist control participants with MS.

##### Hypothesis 1.1:

Participants in the home-based M2M teleexercise training program will achieve similar gains in health and function outcomes as the onsite M2M exercise training program.

##### Hypothesis 1.2:

There will be no difference in adverse side effects (*safety*) between the home-based and onsite exercise treatment groups.

## Results

### Overview of recruitment

**Fig. 2:**
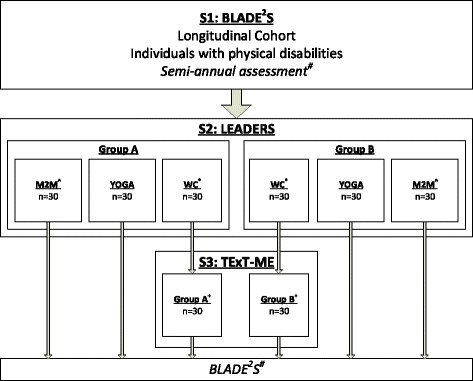
Example of Participant Flow in Russian doll Study Design. # - All participants in the project will continue to receive two assessments annually. ^ - M2M = Movement to Music. * - WC = Waitlist Control. + − The most effective intervention between M2M and Yoga in S2 will be assigned as the intervention in S3 by disability

Figure 2 outlines the flow of participants from enrollment in S1 BLADE^2^S to randomization into S2 and S3. The longitudinal cohort includes individuals who have a neurologic disability. The primary target groups for our first set of exercise interventions include individuals with multiple sclerosis (MS) and stroke, and thus are displayed as those randomized into S2 and S3. One hundred and twenty nine individuals with neurologic disability have been recruited into S1 BLADE^2^S over a period of 24 months (October 2014 to September 2016). Of those individuals, 91 have met the additional criteria for S2 LEADERS and have been randomized into the 3 arms of the intervention. Currently, 28 individuals are eligible for the S3 TExT-ME intervention.

## Discussion

The lack of data pertaining to the frequency, intensity, duration, and modality components of an exercise prescription for people with neurologic disability has limited the utility of exercise as a viable treatment for targeting underperforming physiological (e.g., cardiorespiratory) and neuromotor (e.g., gait, balance) systems and symptom-specific conditions (e.g. pain, fatigue). The Russian doll framework is ideal for using the longitudinal data obtained in BLADE^2^S (S1) to design clinical and home-based exercise trials (LEADERS & TExT-ME). As a group, these three studies will a) help build a comprehensive and sustainable research framework that increases understanding of the onset and course of secondary conditions in people with neurologic disability; b) examine the potential impact that certain types and doses of exercise have on improving health and function; and c) build the infrastructure for supporting safe and effective home-based exercise using telehealth monitoring technologies that can reach geographically or economically isolated populations who have limited or no access to community-based exercise facilities. Moreover, having this infrastructure will lay the foundation for an effective and sustainable approach for onsite and remote delivery of novel exercise protocols that can scaled to other facilities and homes nationwide.

Understanding the nature of complex interactions between the individual and his/her environment is one of the major challenges faced by clinicians, researchers, public health interventionists and policymakers [46–48]. Our first research study, BLADE^2^S, aims to bring greater clarity to the disparate rates of lower health and function in our cohort, in addition to establishing a framework for conducting separate sub-studies examining risk factors and consequences of physical inactivity on key health outcomes.

The selection of multiple groups of people with neurologic disability provides the research team with the opportunity to examine the onset and impact of secondary health conditions and symptoms *between* and *within* neurologic disability groups. Between group comparisons can provide valuable insight into the extent to which specific interventions may be effective in addressing specific secondary conditions across disability groups, while within group comparisons will offer a deeper understanding of the stage and severity of the disability on selection of effective intervention strategies. Both types of comparisons are needed to gain a better understanding of common patterns of health and functional deficits, and to allow researchers to begin developing an evidence base for the most effective and cost-effective interventions.

In the future, BLADE^2^S will allow us to establish normative values for clinical and functional assessments and validate assessment tools for adults with neurologic disability, and track changes on a variety of variables and their associations/effects on indices of health, function, fitness, and quality of life. We anticipate that the additional exploratory analyses derived from BLADE^2^S data will generate a myriad of hypotheses that can inform intervention development and delivery, service provision, and the management of risk factors for people with neurologic disability. As our cohort increases in sample size across different neurologic disabilities, we will be able to implement the Russian doll study framework in a cohort multiple randomized controlled trial [49]. This will enable us further to implement studies using a patient-centered approach.

The second part of our Russian doll framework (LEADERS) is used to develop clinical exercise training studies that address identified health and functional deficits. Data on exercise dose-response are critical for understanding and providing appropriate, targeted interventions that can reduce health and functional deficits and improve symptom management. LEADERS uses specific doses of exercise to determine their potential effect on improving health and function in people with a range of neurologic disability. In the future, the LEADERS framework will be able to serve as a ‘hub’ for researchers to design and test certain types of innovative exercise interventions hypothesized to be beneficial for addressing certain health and functional deficits in different subgroups of adults with neurologic disability.

Our third study, TExT-ME, is testing the feasibility and effectiveness of transferring successful clinical interventions into the home setting using the latest innovative telehealth technology, which includes a home exercise training and monitoring platform to conduct high fidelity, safe and effective dose-response training studies. Conducting home-based exercise trials was historically a problem because of the limited capacity in monitoring participants for safety; not being able to determine if participants were adherent to the training protocol; and having limited capacity to provide personal and motivational support for the participant during exercise. However, recent developments in new monitoring technologies provide opportunities to deliver safe exercise recommendations remotely. The benefits of teleexercise also include reducing the barrier of transportation, offering participants the flexibility of exercising at their preferred time of day, and not requiring as much energy or time getting to an exercise facility. It is also an excellent way to begin encouraging people with neurologic disability who have concerns or fears about exercising in public settings (i.e., fitness facilities) to build confidence during the early stages of an exercise intervention by learning appropriate exercise techniques and improving their exercise self-efficacy. The implication of the TExT-ME study is that once the intervention is found to be safe and effective, we will be able to deliver remote home-based exercise training protocols to a variety of hard-to-reach, geographically isolated populations where access to community-based exercise facilities are limited or non-existent.

## Conclusion

Knowledge of the prevalence of various health behaviors and their potential impact on health and function is critical for planning appropriate interventions that address important health issues underdiagnosed and/or untreated in people with neurologic disability. Our three-in-one Russian doll prospective cohort study framework allows us to explore the interaction of sociodemographic factors, secondary health conditions, lifestyle health behaviors, and the built environment on health trajectories in a cohort of adults with neurologic disability, followed by two intervention study designs examining the effects of clinic and home exercise treatments. To further delineate the complex interaction between disability, health behaviors and the environment, we are documenting changes in health and function outcomes including quality of life, and are exploring independent predictors and associations between these three constructs (health, behavior & environment). We also will determine if exercise participation and changes to health and function outcomes are obtained in one or both settings.

The development of innovative strategies for tracking and improving health, preventing or minimizing secondary conditions associated with neurologic disability, and providing clinicians and health professionals with evidence-based exercise guidelines for managing the health and accommodating secondary conditions of their patient/client population is a new generation of research that must be recognized as a high priority for adults with neurologic disability.

## Abbreviations

BLADE^2^S: Birmingham-Lakeshore Aging with Disability Exercise-Environment Study
DXA: Dual-energy X-ray absorptiometry
LEADERS: Lakeshore Examination of Activity, Disability, and Exercise Response Study
M2M: Movement-to-Music
MS: Multiple Sclerosis
PROMIS: Patient-Reported Outcomes Measurement Information System
QOL: Quality of Life
S1: Study 1
S2: Study 2
S3: Study 3
TExT-ME: Telehealth Exercise Training - Monitoring and Evaluation
UCD: User centered design
